# Health literacy and adherence to physical activity guidelines among adults with hypertension: evidence from a Korean National Survey

**DOI:** 10.1016/j.pmedr.2025.103260

**Published:** 2025-10-05

**Authors:** Eun Jung Bae, Joohyun Chung, Heesook Son

**Affiliations:** a Department of Nursing, Ansan University, Republic of Korea; b College of Nursing, University of Massachusetts, 651 N Pleasant St, Amherst, MA 01003, USA; c Red Cross College of Nursing, Chung-Ang University, 84 Heukseok-ro, Dongjak-gu, Seoul 06974, Republic of Korea

**Keywords:** Health literacy, Hypertension, Physical activity adherence, Preventive health behavior, Behavioral determinants

## Abstract

**Objective:**

Hypertension is a major contributor to premature mortality and global disease burden. Physical activity is a key non-pharmacological management strategy; however, adherence remains low. Health literacy may influence health behaviors, yet its association with physical activity among individuals with hypertension is unclear. This study examined the relationship between health literacy and physical activity guideline adherence in Korean adults with hypertension using nationally representative data.

**Methods:**

We conducted a secondary analysis of the 2023 Korea National Health and Nutrition Examination Survey using multistage stratified cluster sampling. Among 1420 adults with hypertension, health literacy was assessed with a 10-item scale, with scores ≥30 indicating adequacy. Physical activity adherence followed World Health Organization guidelines: at least 150 min of moderate, 75 min of vigorous activity, or an equivalent combination per week. Complex sample logistic regression analyses were performed, adjusting for sociodemographic, health, behavioral, and psychosocial covariates.

**Results:**

Adults with adequate health literacy had significantly higher odds of meeting recommended activity levels. Younger age, higher education, better self-rated health, and absence of activity restriction were also positively associated.

**Conclusions:**

Health literacy is a key determinant of physical activity in adults with hypertension. Enhancing health literacy may improve hypertension management.

## Highlights

• Hypertension is a public health concern affecting 33 % of adults worldwide.
• This study examined physical activity in Korean adults with hypertension.
• Health literacy was associated with adherence to physical activity guidelines.
• Improving health literacy could help increase physical activity in this population.

## 1 Introduction

Hypertension is a major public health concern, affecting approximately 33 % of adults aged 30–79 years worldwide (World Health Organization (WHO), 2023). It remains the leading cause of early mortality and accounts for 7.8 % of all global disability-adjusted life years (DALYs; GBD 2021 Risk Factors Collaborators, 2024; Institute for Health Metrics and Evaluation, 2024). This trend is also evident in Korea, where the prevalence of hypertension was 23.4 % among adults aged ≥19 years and 29.5 % among those aged ≥30 years in 2023 (Korea Disease Control and Prevention Agency [KDCA], 2024a).

Participation in physical activity remains suboptimal among Korean adults with hypertension despite clear evidence supporting its benefits in hypertension management. Only 40.1 % of Korean adults with hypertension engage in sufficient physical activity (Eom et al., 2025). WHO guidelines recommend at least 150 min of moderate-intensity or 75 min of vigorous-intensity aerobic activity per week or an equivalent combination (Bull et al., 2020). Aerobic exercise effectively lowers blood pressure, with evidence supporting a dose-dependent reduction in blood pressure, particularly at the 150-min threshold (Carey et al., 2022; Jabbarzadeh Ganjeh et al., 2024). Regular physical activity not only lowers blood pressure but also reduces the risk of hypertension-related complications, including ischemic heart disease, heart failure, and chronic kidney disease. By preventing or delaying these conditions, physical activity contributes to reducing long-term disease burden and DALYs (Xu et al., 2022). Nevertheless, individuals with hypertension often face difficulties initiating and sustaining regular physical activity. Reported barriers include low self-efficacy, limited social support, competing time demands, socioeconomic constraints, and the absence of enjoyable or tailored exercise options, all of which hinder adherence and long-term maintenance (Lopes et al., 2021). This highlights the importance of identifying modifiable factors that facilitate physical activity adherence.

Health literacy, defined as the ability to access, understand, appraise, and apply health information, is increasingly recognized as a foundational element of health promotion and a critical determinant of health behavior (Sørensen et al., 2012). Global strategies have emphasized enhanced health literacy as a key target for improving population health and managing chronic diseases (Okan et al., 2019). Various approaches to enhancing health literacy in individuals with chronic conditions have been proposed, including simplified educational materials, pictorial aids, and the teach-back method (Ha Dinh et al., 2016). Structured programs combining active learning and peer support have also demonstrated effectiveness in promoting health literacy and improving health behaviors in older adults (Uemura et al., 2018).

Previous studies have demonstrated a positive association between health literacy and physical activity in the general population (Buja et al., 2020). However, this relationship cannot be assumed to operate similarly in individuals with hypertension, who often face unique barriers, including concerns about overexertion, uncertainty regarding safe activity levels, and comorbidities that complicate behavioral decisions (Vieira et al., 2024). Despite these challenges, research directly examining this link in hypertensive populations remains limited (Aaby et al., 2017). Our study addresses this gap using the most recent nationally representative data from the 2023 Korea National Health and Nutrition Examination Survey (KNHANES), which incorporated a newly validated health literacy measure (Yoon et al., 2023). Notably, hypertension disproportionately affects individuals with limited health literacy (Sohrabi et al., 2022), underscoring the relevance of this study in addressing health disparities.

Furthermore, as a modifiable and actionable construct, health literacy represents a promising entry point for health promotion interventions aimed at improving physical activity adherence in this population. The well-documented benefits of physical activity for blood pressure (Jabbarzadeh Ganjeh et al., 2024) support the need to improve the current understanding of how health literacy influences physical activity engagement in adults with hypertension. Therefore, this study used nationally representative data to examine this association and inform more effective hypertension management strategies.

## 2 Methods

### 2.1 Data source and study population

This study was a secondary data analysis using the 2023 KNHANES, conducted by the KDCA. The KNHANES is a national survey designed to monitor the health and nutritional status of Korean population and to provide data for evaluating the National Health Promotion Plan and informing public health policies. It collects comprehensive information on health statis, health behaviors, and dietary and nutritional intake through health interviews, nutrition surveys, and health examinations. The survey employs a two-stage stratified cluster sampling design based on the most recent Population and Housing Census to ensure national representativeness (KDCA, 2024b). In the first stage, 192 survey districts were selected, followed by 25 households per district. All household members aged ≥1 year who met the eligibility criteria were included. The 2023 survey comprised 6929 respondents. Detailed information on data collection and processing is publicly available.

This study initially identified 1683 adults aged ≥19 years who were diagnosed with hypertension by a physician. Although hypertension is often associated with older adults, recent data show a growing prevalence in younger individuals, including those in their 20s (Hinton et al., 2020). To reflect this trend, adults aged 19–29 years were also included to capture the burden of early onset hypertension and its implications for preventive behavior. Respondents who were not receiving treatment (*n* = 99) or did not respond to questions regarding aerobic exercise (*n* = 164) were excluded, resulting in a final sample of 1420 adults with hypertension.

### 2.2 Measures

#### 2.2.1 Physical activity

The KNHANES assesses physical activity using the Global Physical Activity Questionnaire, which measures the intensity, frequency, and duration of activity across occupational, transportation, and leisure domains. Activity lasting at least 10 min is recorded, with moderate intensity defined as inducing a slight increase in breathing and heart rate, and vigorous intensity as causing a significant increase (KDCA, 2024a).

In this study, physical activity adherence was defined according to international guidelines as engaging in at least 150 min of moderate-intensity activity, 75 min of vigorous-intensity activity, or an equivalent combination per week, with 1 min of vigorous activity considered equivalent to 2 min of moderate activity (Bull et al., 2020). Participants meeting this criterion were classified as adherent, whereas others were classified as non-adherent.

#### 2.2.2 Health literacy

Health literacy was assessed using a 10-item instrument developed to monitor health literacy in the general population, which was first added to the KNHANES in 2023 (Yoon et al., 2023). The tool includes three items on disease prevention, one on health promotion, four on health management, and two on resource utilization, rated on a 4-point Likert scale (see Appendix). Reliability was high, with a Cronbach's α of 0.87 overall and 0.72–0.78 across subdomains (Yoon et al., 2023). According to KDCA guidelines, a total score ≥ 30 indicates adequate health literacy (KDCA, 2024a). Participants were classified as having adequate or inadequate health literacy based on this cutoff point.

#### 2.2.3 Covariates

Sociodemographic variables included sex, age, educational attainment, income, and marital status. Health-related covariates were selected based on prior evidence linking health conditions to physical activity (Lim and Yoo, 2020). These included subjective health status, diabetes, dyslipidemia, body mass index (BMI)-based weight status, and activity restriction. Diabetes and dyslipidemia were defined as physician-diagnosed and currently treated conditions. BMI was categorized according to the WHO Asia-Pacific classification, with obesity defined as BMI ≥ 25 kg/m^2^. This classification was chosen instead of the standard WHO cut-offs because it better reflects the higher metabolic and cardiovascular risks observed at lower BMI thresholds in Asian populations (WHO Expert Consultation, 2004). Activity restriction refers to self-reported limitations in daily or social activities because of physical or mental health problems. Considering the clustering of health behaviors (Rabel et al., 2018), smoking, alcohol use, sleep duration, and breakfast frequency were also included. In addition, perceived stress and depressive symptoms were included as psychological covariates because of their bidirectional relationship with physical activity (Stults-Kolehmainen and Sinha, 2014). Perceived stress was classified based on participants' response in daily life: those who reported feeling “very much” or “much” stress were categorized as having high perceived stress, whereas those who responded “a little” or “hardly any” were classified as having low perceived stress. The variable of depressive symptoms was categorized according to whether participants had felt depressed for at least two consecutive weeks during the past year to the extent that it interfered with their daily life.

### 2.3 Statistical analysis

To address this study's aims, descriptive statistics were used to summarize participant characteristics, and the Rao–Scott χ^2^ test was used to assess differences in physical activity adherence across sociodemographic, health, behavioral, and psychosocial variables. Complex sample logistic regression was performed to examine the association between health literacy and physical activity adherence, and unadjusted and adjusted odds ratios (ORs) were reported with 95 % confidence intervals (CIs), with adjusted estimates controlling for covariates. The KNHANES employs a complex sampling design involving stratification, clustering, and multistage sampling; thus, sample weights, strata, and clusters were applied to account for the design and ensure national representativeness (KDCA, 2024b). Statistical significance was set at *p* < .05. All analyses were conducted using the Complex Samples module in SPSS (version 26.0; IBM Corp., Armonk, NY).

### 2.4 Ethical considerations

This study was granted an exemption from review by the Public Institutional Bioethics Committee (IRB No. P01-202504-01-014).

## 3 Results

### 3.1 Participant sociodemographic, health, behavioral, and psychosocial characteristics

Table 1 summarizes the participant characteristics (*N* = 1420). The sample was 52.1 % male, with the largest age group being ≥70 years. Overall, 25.7 % of the respondents had diabetes, 53.5 % had dyslipidemia, 49.4 % were obese, and 12.5 % reported activity restriction. Good or very good subjective health was reported by 28.1 % of the sample. Regarding behaviors, 47.0 % were current smokers and 47.0 % consumed alcohol monthly. In terms of health literacy and behavior, 48.7 % scored ≥30, and 37.6 % met physical activity guidelines. Health literacy was analyzed as a binary variable (adequate vs. inadequate) based on the KDCA threshold (≥ 30 points).

**Table 1:** Sociodemographic, health, behavioral, and psychosocial characteristics of Korean adults with hypertension (*N* = 1420), Korea National Health and Nutritional Examination Survey 2023.

	Variables	Category	n ^a^ (%)^b^
Sociodemographic factors			
	Sex	Male	681 (52.1)
		Female	739 (47.9)
	Age (years)	19–49	96 (9.7)
		50–59	250 (22.7)
		60–69	483 (31.4)
		≥ 70	591 (36.2)
	Educational attainment	≤ Middle school	707 (44.8)
		High school	422 (32.1)
		≥ College	271 (23.1)
	Income	Low	354 (24.2)
		Lower-middle	379 (26.0)
		Upper-middle	347 (24.6)
		High	339 (25.2)
	Marital status	Single	46 (4.6)
		Living with spouse	1030 (73.3)
		Divorced or widowed	331 (22.1)

Health conditions			
	Self-rated health	Good/very good	406 (28.1)
		Fair	691 (48.4)
		Bad/very bad	323 (23.5)
	Diabetes	Yes	375 (25.7)
		No	1045 (74.3)
	Dyslipidemia	Yes	760 (53.5)
		No	660 (46.5)
	Weight status	Underweight/normal weight	369 (25.5)
		Overweight	338 (25.1)
		Obese	665 (49.4)
	Activity restriction	Yes	192 (12.5)
		No	1208 (87.5)

Health-related behaviors			
	Current smoking	Yes	623 (47.0)
		No	783 (53.0)
	Alcohol consumption	None or < 1 drink	783 (53.0)
	(per month)	≥ 1 drink	623 (47.0)
	Sleeping duration	< 7	674 (48.6)
	(hours/day)	≥ 7	725 (51.4)
	Breakfast frequency	Regular	1137 (76.5)
		Irregular	270 (23.5)

Psychological factors			
	Perceive stress	High	265 (20.3)
		Low	1139 (79.7)
	Depressive symptoms	Yes	161 (11.2)
		No	1243 (88.8)

Health literacy		Inadequate (〈30)	760 (51.3)
		Adequate (≥ 30)	660 (48.7)

Physical activity		Adherent	509 (37.6)
		Non-adherent	911 (62.4)

Note: ^a^Unweighted; ^b^Weighted; Subdomain-level health literacy scores: disease prevention (M = 2.91 ± 0.03), health promotion (M = 3.00 ± 0.04), health management (M = 3.08 ± 0.03), and resource utilization (M = 2.81 ± 0.03), all rated on a 4-point Likert scale.

### 3.2 Differences in physical activity guideline adherence by participant characteristics

Table 2 summarizes the differences in aerobic physical activity according to participant characteristics. Higher physical activity adherence was significantly associated with adequate health literacy (*p* < .001), male sex, younger age, higher education, and being married (all *p* < .01). Participants with good self-rated health, no activity restriction, and no diabetes were also more likely to meet the physical activity guidelines (all *p* < .05). Among health behaviors, only alcohol use was significantly associated with activity adherence (*p* = .025).

**Table 2:** Differences in adherence to physical activity guidelines by sociodemographic, health, behavioral, and psychosocial characteristics among Korean adults with hypertension, Korea National Health and Nutritional Examination Survey 2023.

	Variables	Category	Physical Activity		
			Adherent (*n* = 509)	Non-adherent (*n* = 911)	*p*^c^
			n ^a^ (%)^b^	n ^a^ (%)^b^	
Sociodemographic factors					
	Sex	Male	273 (57.9)	408 (48.6)	0.006
		Female	236 (42.1)	503 (51.4)	
	Age (years)	19–49	52 (14.3)	44 (6.9)	<0.001
		50–59	98 (24.6)	152 (21.6)	
		60–69	195 (34.9)	288 (29.3)	
		≥ 70	164 (26.3)	427 (42.2)	
	Educational attainment	Middle school or lower	203 (34.6)	504 (50.9)	<0.001
		High school	159 (32.5)	263 (31.8)	
		College or higher	141 (32.9)	130 (17.2)	
	Income	Low	118 (22.3)	236 (25.4)	0.185
		Lower-middle	131 (24.7)	248 (26.8)	
		Upper-middle	125 (24.3)	222 (24.8)	
		High	135 (28.8)	204 (23.0)	
	Marital status	Single	20 (5.6)	26 (3.9)	<0.001
		Married	409 (80.4)	621 (68.9)	
		Divorced or widowed	79 (13.9)	252 (27.1)	

Health conditions					
	Self-rated health	Good/very good	142 (29.0)	181 (20.1)	<0.001
		Fair	255 (49.4)	436 (47.8)	
		Bad/very bad	112 (21.6)	294 (32.1)	
	Diabetes	Yes	122 (21.9)	253 (28.0)	0.016
		No	387 (78.1)	658 (72.0)	
	Dyslipidemia	Yes	272 (53.2)	488 (53.7)	0.870
		No	237 (46.8)	423 (46.3)	
	Weight status	Underweight/normal weight	141 (25.8)	228 (25.3)	0.976
		Overweight	120 (24.8)	218 (25.3)	
		Obese	237 (49.4)	428 (49.5)	
	Activity restriction	Yes	47 (8.2)	145 (15.0)	<0.001
		No	456 (91.8)	752 (85.0)	

Health-related behaviors					
	Current smoking	Yes	79 (17.0)	120 (15.5)	0.540
		No	424 (83.0)	779 (84.5)	
	Alcohol consumption	None or < 1 drink	252 (48.4)	531 (55.8)	0.025
	(per month)	≥ 1 drink	253 (51.6)	370 (44.2)	
	Sleeping duration	< 7	240 (48.2)	434 (48.8)	0.856
	(hours/day)	≥ 7	263 (51.8)	462 (51.2)	
	Breakfast frequency	Regular	403 (75.1)	734 (77.4)	0.404
		Irregular	101 (24.9)	169 (22.6)	

Psychological factors					
	Perceive stress	Much	92 (18.8)	173 (21.2)	0.320
		Less	411 (81.2)	728 (78.8)	
	Depressive symptoms	Yes	48 (9.8)	113 (12.0)	0.297
		No	455 (90.2)	788 (88.0)	

Health literacy		Inadequate (< 30)	230 (42.1)	530 (56.9)	<0.001
		Adequate (≥ 30)	279 (57.9)	381 (43.1)	

Note: ^a^Unweighted; ^b^Weighted; ^c^p-values for testing differences using a Rao–Scott χ^2^ test.

### 3.3 Association between health literacy and physical activity guideline adherence among adults with hypertension

Table 3 presents the results of the complex sample logistic regression analysis examining the association between health literacy and adherence to physical activity guidelines among adults with hypertension. Adults with a health literacy score ≥ 30 were significantly more likely to meet the recommended level of physical activity than those with lower scores (unadjusted OR = 1.76, 95 % CI: 1.42, 2.16). This association remained significant after controlling for covariates (adjusted OR = 1.28, 95 % CI: 1.01, 1.63).

**Table 3:** Association between health literacy and adherence to physical activity guidelines among Korean adults with hypertension, Korea National Health and Nutritional Examination Survey 2023.

Variables		Category	Unadjusted OR (95 % CI)	Adjusted OR (95 % CI)
Health literacy		Adequate (≥ 30)	1.76 (1.42, 2.16)	1.28 (1.01, 1.63)
		Inadequate (< 30)	1.00	1.00

Note: Control variables: sex, age, educational attainment, marital status, self-rated health, diabetes, activity restriction, and alcohol consumption; OR: Odds Ratio; CI: Confidence Interval.

## 4 Discussion

This study used nationally representative data to examine the association between health literacy and physical activity adherence in adults with hypertension. Descriptive analysis showed that 48.7 % of the participants had adequate health literacy, which is lower than the 2023 national average of 60.4 % for adults aged ≥19 years (KDCA, 2024a). This gap likely reflects the older age profile of the population with hypertension. National data indicate that health literacy declines sharply with age, from 70.5 % in adults aged 19–29 years to 36.0 % in those aged ≥70 years (KDCA, 2024a). These findings suggest that age composition may partially explain the lower level of health literacy observed in this sample of adults with hypertension.

This study found that adults with hypertension who had adequate health literacy were significantly more likely to meet the recommended physical activity levels than those with inadequate health literacy. Regular physical activity is a well-established non-pharmacological strategy for hypertension management that can reduce blood pressure and prevent cardiovascular complications (Carey et al., 2022). Although prior studies have linked health literacy to positive health behaviors in the general population (Buja et al., 2020), this relationship in people with chronic conditions such as hypertension is more complex and inconsistent.

Unlike healthy adults, individuals with hypertension face unique challenges that can weaken the link between knowledge and behavior. These include concerns about exercise risk and uncertainty regarding appropriate activity levels. Furthermore, comorbidities may complicate decision-making (Vieira et al., 2024). Inconsistent findings in populations with chronic diseases may be explained by depressive symptoms and socioeconomic barriers. These factors can limit behavior change, even when individuals have sufficient health knowledge (Osborn et al., 2011). Considering these complexities, confirming this positive association supports the need for tailored interventions. Enhancing health literacy may be a key strategy for promoting physical activity in this high-risk population, for whom behavior change is often hindered by multiple barriers. Recent evidence supports the effectiveness of theory-based behavior change interventions in modifying lifestyle risk factors for non-communicable diseases, particularly when interpersonal and integrated approaches are applied (Darukaradhya and Krishnamurthy, 2025). Similarly, studies in occupational groups, such as teachers, have shown that an unhealthy diet and sedentary lifestyle are significant contributors to hypertension prevalence (Kumar et al., 2025). Moreover, gaps in awareness, treatment, and adherence, often described in relation to the “rule of halves,” continue to challenge hypertension management in low- and middle-income settings (Shivani et al., 2024). Together, these findings highlight the importance of integrating behavior change strategies and health literacy enhancement in hypertension care.

Disease-specific knowledge is a key mechanism linking health literacy to behavior. For patients with hypertension, knowing that physical activity helps control blood pressure can reframe exercise as part of their treatment rather than as a risk (Barone Gibbs et al., 2021). Studies have shown that lower health literacy is associated with limited knowledge and poor self-management in people with hypertension (Osborn et al., 2011; van der Heide et al., 2014). Therefore, enhancing health literacy may improve condition-specific understanding and support informed decisions regarding health behaviors, including physical activity.

Self-efficacy is another key psychological factor supporting self-management in chronic conditions (Bandura, 1977). Lower self-efficacy is a known barrier to effective self-care and behavior change in hypertension (Murphy et al., 2015; Tan et al., 2021). Recent studies have indicated that health literacy may foster self-efficacy, particularly in chronic disease management (Kaplan et al., 2024). This suggests that health literacy may promote adherence by increasing confidence in managing health behaviors.

This study adds to the growing evidence that health literacy is a key determinant of health promotion in chronic disease management. Although the association observed in this study was statistically significant, the effect size was modest. Nevertheless, these findings are meaningful. Health literacy may provide a practical target for interventions aimed at improving hypertension management. Improving health literacy could yield small but clinically relevant gains in physical activity, thereby supporting improved blood pressure control and lowering cardiovascular risk. At the population level, even modest individual effects may accumulate and produce substantial public health benefits, particularly considering the high prevalence of hypertension. By focusing on adults with hypertension, this study clarified the role of health literacy in supporting physical activity adherence. Considering these findings, interventions should enhance health literacy, particularly in terms of skills to help people understand, evaluate, and apply health information to sustain behavior change.

Although this study provides important insights using nationally representative data, several limitations should be noted. First, its cross-sectional design precludes causal inferences. Second, both health literacy and physical activity were assessed via self-report, which may have introduced recall bias, social desirability bias, and measurement validity concerns. However, the health literacy instrument used in this study was specifically developed and validated for national use in Korea (Yoon et al., 2023), and the physical activity assessment followed the WHO-recommended Global Physical Activity Questionnaire (Bull et al., 2020). Nevertheless, the potential for misclassification or bias cannot be ruled out. Third, the absence of key mediating variables, such as motivation, disease-specific knowledge, and self-efficacy, limits the ability to determine whether the observed association reflects a direct effect of health literacy. Furthermore, whether the effect operates through indirect pathways involving cognitive, emotional, or behavioral processes remains unclear. This gap also constrains the identification of specific causal components that could otherwise be targeted to enhance intervention effectiveness.

Despite these limitations, this study has several notable strengths. It used a large, nationally representative sample of Korean adults with hypertension, thus enhancing the generalizability of the findings to the target population. The complex, multistage stratified sampling design of the KNHANES and the application of appropriate analytical weights ensured that the estimates accurately reflected the population parameters. Both the health literacy instrument and physical activity assessment tool were validated in the Korean context, supporting measurement reliability and cultural relevance. Furthermore, by focusing specifically on adults with hypertension, this study provides evidence directly applicable to clinical and public health strategies for this population with elevated cardiovascular risk. These findings also offer practical implications for policy and intervention design, particularly for developing age-sensitive and literacy-informed approaches to improve physical activity adherence.

## 5 Conclusion

This study highlights the role of health literacy in promoting physical activity adherence among adults with hypertension. Tailored strategies, such as simplified materials, pictorial aids, and the teach-back method, are recommended to reduce behavioral disparities, especially in older and socioeconomically disadvantaged groups (Ha Dinh et al., 2016). Structured group-based programs that combine active learning and peer support may further enhance both health literacy and physical activity engagement (Uemura et al., 2018). Implementing these strategies in hypertension care could improve behavioral adherence and promote health equity.

Future research should incorporate objective measures, such as accelerometers and performance-based health literacy assessments, to improve measurement precision and validity. In addition, because of data limitations, this study did not examine potential mediating or moderating mechanisms, such as self-efficacy, disease-specific knowledge, or motivational readiness. The 2023 KNHANES health literacy module did not include these constructs, precluding further exploration of behavioral pathways. However, these psychological factors likely play a pivotal role in translating health literacy into sustained physical activity. Future studies should address these gaps using longitudinal or intervention-based designs combined with more nuanced statistical modeling, such as mediation and moderation analyses, to clarify the mechanisms linking health literacy to physical activity adherence. In particular, research should investigate the roles of self-efficacy, disease knowledge, and motivation, and consider sociodemographic and cultural factors to identify vulnerable subgroups and guide the development of tailored health promotion strategies.

## CRediT authorship contribution statement

**Eun Jung Bae:** Writing – review & editing, Writing – original draft, Methodology, Formal analysis, Data curation, Conceptualization. **Joohyun Chung:** Writing – review & editing, Writing – original draft, Methodology, Formal analysis. **Heesook Son:** Writing – review & editing, Writing – original draft, Supervision, Methodology, Funding acquisition, Conceptualization.

## Patient consent statement

This study was based on a secondary analysis of publicly available, de-identified data from the Korea National Health and Nutrition Examination Survey (KNHANES). As the dataset contains no personally identifiable information, the requirement for individual informed consent was waived.

## Funding

This work was supported by a 10.13039/501100003725National Research Foundation of Korea Grant funded by the South Korean Government (NRF-2022R1F1A1068236) and 10.13039/501100002460Chung-Ang University Research Grants in 2023.

## Declaration of competing interest

The authors declare that they have no known competing financial interests or personal relationships that could have appeared to influence the work reported in this paper.

## Data Availability

The data used in this study are publicly available from the Korea Disease Control and Prevention Agency (KDCA). Detailed information about the KNHANES survey and instructions for data access are available at https://knhanes.kdca.go.kr/knhanes
